# A window of opportunity? Motor skills and perceptions of competence of children in Kindergarten

**DOI:** 10.1186/1479-5868-9-29

**Published:** 2012-03-15

**Authors:** Mark LeGear, Lizette Greyling, Erin Sloan, Rick I Bell, Buffy-Lynne Williams, Patti-Jean Naylor, Viviene A Temple

**Affiliations:** 1 School of Exercise Science, Physical and Health Education, University of Victoria, Victoria, Canada; 2 School Age Program, Queen Alexandra Centre for Children's Health, Victoria, Canada

**Keywords:** Motor skills, Perceptions of Competence, Children, Kindergarten, Gender

## Abstract

**Background:**

Our aim was to examine the relationship between motor skill proficiency and perceptions of competence of children in their first year of school. We also explored gender-based differences.

**Findings:**

Participants were 260 kindergarten children (mean age = 5y 9 m; boys = 52%) from eight schools; representing 78% of eligible children in those schools. Motor skills were measured using the Test of Gross Motor Development-2 and perceptions of physical competence were assessed using the Pictorial Scale of Perceived Competence and Social Acceptance for Young Children. Motor skill scores were generally low (percentile ranks ranged from 16 - 24) but perceptions of physical competence were positive (boys = 18.1/24.0, girls = 19.5/24.0). A MANOVA showed a significant overall effect for gender (Wilk's lambda = .84 with *F *(3, 254) = 15.84, *p *< 0.001) and univariate F tests were significant for all outcome variables. The relationship between object control skills and perceptions of physical competence among girls was not significant; however all other correlations were modest but significant.

**Conclusions:**

Although motor skill levels were quite low, the children generally held positive perceptions of their physical competence. These positive perceptions provide a window of opportunity for fostering skillfulness. The modest relationships between perceptions of competence and motor skill proficiency suggest that the children are beginning to make self-judgments at a young age. Accordingly, opportunities for children to become and feel physically competent need to occur early in their school or preschool life.

## Background

Only 9% of boys and 4% of girls meet the Canadian Physical Activity Guidelines of 60-minutes of moderate-to-vigorous physical activity on at least six days a week [1]. These alarming data suggest there is an urgent need to examine factors associated with children's inactivity. Emerging evidence suggests that motor skill competence as well as children's perceptions of their competence are influential in explaining their engagement and disengagement in physical activity. Barnett and colleagues [2,3] demonstrated that object control motor skills measured at age 10 were positively associated with adolescents' physical activity. This is consistent with cross-sectional findings showing that motor skill proficiency is associated with participation in organized sport [4], skill specific physical activity [5], and participation in physical activity [6-9]. Research also suggests that poor motor skills are associated with lower perceptions of physical competence [8-12]. Recent findings point toward a mediating role between children's perceptions of competence and engagement in physical activity [13,14]. For example, Barnett and others found that physical activity levels of Grade 10 students were associated with childhood proficiency with object control skills; but proficiency was mediated by their perceptions of sports competence.

Stodden and colleagues [15] modeled some of this complexity. These authors proposed that motor competence, not just perceptions of motor competence, was at the heart of a developmental model that might explain participation in physical activity. They suggested that children with poor motor competence have low self-efficacy toward participation. Low self-efficacy subsequently mediates perceptions of competence, which in turn, influences the child's desire to engage with physical activity. Additionally, these perceptions of competence change from early to middle childhood. Young children tend to have inflated perceptions of their motor competence [16] as they do not possess the cognitive skills to distinguish between actual competence and effort [17,18]. These inflated perceptions are potentially valuable because they present a window of opportunity where young children may persist with, and attempt to master, activities they believe they're good at [15,17]. Evidence shows that intervention at this young age can produce significant and meaningful improvements in motor skill proficiency [19-21] and positively influence perceptions of competence and self-esteem [22].

The potential importance of children's perceptions of competence as a predictor or mediating variable led us to examine the relationship between motor skill proficiency and perceptions of competence of children in their first year of school. We also explored gender-based differences. Both physical activity levels and gross motor skill competence have been shown to be influenced by gender. Boys tend to be more active [23-25] and have more developed object control skills than girls [14,18,26-28]. However, boys' locomotor proficiency has been reported as lower [14,27], equivalent to [18], or higher [26] than girls. Among young children, perceptions of physical competence have been reported as equivalent between the genders [17,18] or more favorable among boys [29]. Consistent with Stodden and colleagues' model [15], we hypothesized that the children would exhibit high levels of perceived physical competence, but there would be no relationship between perceptions of physical competence and motor skill proficiency. Further, we hypothesized that boys would show greater motor skill proficiency than girls.

## Methods

### Participants

Kindergarten children (*n *= 267) from eight schools in one school district in British Columbia, Canada were recruited. Rates of vulnerability among kindergarten children in the Province of British Columbia have been mapped using the Early Development Index (EDI) since 2000 [30]. For the school district in this study, the most recent EDI data (2009 - 2011) reveal rates of vulnerability that are lower than, or equivalent to, provincial rates. Specifically, rates of vulnerability in this school district compared with provincial rates were: Physical Health and Well Being, 13% vs. 13.5%; Social Competence, 15% vs. 14.5%; Emotional Maturity, 13% vs. 13.8%; Language and Cognitive Development, 9% vs. 10.3%; and Communication Skills, 12% vs. 13.7% [31].

Data were incomplete for seven children, therefore the final sample was 260 children (mean age = 5y9m; boys = 52%); representing 78% of the enrolled kindergarten children. Approval for this study was granted by the University Human Research Ethics Board and the school district. Written informed consent was obtained from parents and children provided assent.

### Measures

Fundamental motor skills (six locomotor skills: run, jump, hop, slide, gallop, and leap; and six object control skills: throw, roll, kick, strike, catch, and bounce) were assessed using the Test of Gross Motor Development [TGMD-2; 28]. Children's perceptions of competence were measured using the Pictorial Scale of Perceived Competence and Social Acceptance for Young Children [17]. The scale measures perceptions of cognitive and physical competence as well as peer and maternal acceptance. Only the Perceptions of Physical Competence sub-scale was included in these analyses, with a range of possible scores from 6 - 24. Both tools have acceptable reliability and validity [17,28,32].

### Procedures

Motor skills were assessed during scheduled physical education lessons in accordance with the testing procedure outlined in the TGMD-2 Examiner's Manual [28]. Each class was divided into four small groups (3-5 children). These groups rotated around four stations (3 skills per station). As lessons at each school varied in duration, 1 - 3 lessons were required to gather these data. The performance of consented children was digitally video-recorded. Non-consented children also participated in the skills as per the teachers' and administrators' wishes; and all children participated in games toward the end of each lesson. The Pictorial Scale of Perceived Competence and Social Acceptance for Young Children was administered individually following the physical education lessons.

### Data treatment and analysis

The behavioral components of each skill were scored by the investigators dichotomously; 0 or 1 depending on whether the component was completed correctly by the child. These behavioral components were summed to provide subtest raw scores for locomotor skills (range 0 - 48) and object control skills (range 0 - 48), and a total skills raw score (range 0 - 96). To establish inter-observer reliability, two investigators coded a minimum of 15% of the digital videos (2 - 3 children per class) for each of the 20 kindergarten classes. The Percent Agreement Method [Number of Agreements/(Number of Agreements + Disagreements) × 100] [33] was used to calculate inter-observer reliability between the coders. Percent agreement ranged from 80.2% to 94.8%, with a mean of 87.8%.

Descriptive statistics for the locomotor, object control, and total raw scores were computed and gender- and age-matched normative percentile ranks were computed using the TGMD-2 Examiner's Manual [28]. Averages (range 6-24) were computed for the Perceptions of Physical Competence sub-scale. A multivariate analysis of variance (MANOVA) with gender as a factor and age in months as a covariate was used to analyze the dependent measures: object control skills, locomotor skills, and perceptions physical competence. A separate analysis of covariance for total skills was computed with gender as a factor and age in months as the covariate. Pearson Product-moment correlations were used to examine relationships between the dependent measures. All analyses were performed using SPSS^® ^19 for Windows.

## Results

Descriptive statistics for perceived physical competence and the three motor skill raw scores are shown in Table 1. The mean locomotor raw scores reported in Table 1 translate to percentile ranks of 18 for boys and 24 for girls; and object control raw scores translate to percentile ranks of 18 for boys and 16 for girls. The MANOVA showed a significant overall effect for gender as suggested by Wilk's lambda [34] of .84 with *F *(3, 254) = 15.84, *p *= < 0.001. Results of univariate *F *tests for gender are presented in Table 2. Given the significant gender-based differences, correlations were conducted separately for boys and girls (see Table 3). The analysis of covariance adjusted for age with total skills as the dependent measure showed no difference in fundamental motor skills between boys and girls *F*(1, 258) = .719, *p *= .397.

**Table 1:** Means and SD of perceived competence and raw motor skill scores by gender

	Boys			Girls		
Variable	N	M	SD	N	M	SD
Perceptions of physical competence^a^	135	18.13	3.25	125	19.50	3.05
Locomotor skills^b^	135	25.07	7.38	125	26.87	7.24
Object control skills^b^	135	22.53	7.98	125	19.25	6.06
Total skills^c^	135	47.60	13.86	125	46.12	11.11

Range of possible scores were ^a^6-24, ^b^0 - 48, ^c^0 - 96

**Table 2:** Summary of the univariate *F *tests for perceptions of competence and motor skills with gender as a factor

Variable	*DF*	*MS*	*F*	*p*
Perceptions of physical competence	256	125.49	12.63	< 0.001
Locomotor skills	256	259.44	4.98	0.027
Object control skills	256	646.62	12.72	< 0.001

**Table 3:** Correlations between motor skill scores and perceptions of physical competence by gender

	Perceived Physical Competence		
	All children (n = 260)	Girls (n = 125)	Boys (n = 135)
Total Skills	0.26**	0.33**	0.24**
Object Control Skills	0.14*	0.17	0.21*
Locomotor Skills	0.31**	0.37**	0.22*

** Correlation is significant at the .01 level, * Correlation is significant at the .05 level.

## Discussion

We set out to explore the relationship between motor skill proficiency and perceptions of physical competence of children in kindergarten. The influence of gender on this relationship was also examined. The raw scores reported in Table 1 and the percentile ranks based on Ulrich's [28] normative data illustrate that the gross motor proficiency was generally low. The children in our sample were selected from a school district with low levels of vulnerability; suggesting that these low levels of motor skill proficiency are not related to disadvantage. However, it is possible that the motor skill proficiency of children today is different from Ulrich's normative sample collected during 1997 and 1998 in the USA. Physical activity provides opportunities for neuromotor development [15] and it is feasible that the low levels of physical activity among Canadian children [1] may be hampering this area of development. Notwithstanding the limitations of the normative database, the locomotor and object control raw scores illustrate low levels of fundamental motor skill mastery. The reasons for these low levels of mastery require additional research.

Gender-based differences in motor proficiency were also evident in this study. The differences are in concert with studies showing greater locomotor proficiency among girls [14,27,35] and better object control skills among boys [26,35]. Similar to the findings of Hardy and colleagues [35], we found that combining locomotor and object control skills (i.e. total skills) masked the gender-based differences.

In contrast to the low levels of motor skill proficiency, the mean scores for perceptions of physical competence for both boys and girls were relatively high and consistent with ranges previously reported for kindergarten children [17,18]. A gender-based difference, in favor of the girls, was also evident. No previous studies have found higher perceived physical competence for girls [14,18,26]. One possible explanation is that the assessment of perceived physical competence specifically asks children about running, hopping, skipping, swinging, climbing, and tying shoelaces. Three of these skills are locomotor skills and none of the skills are object control skills. Given the girls' superior locomotor proficiency compared with the boys and compared with their own object control skills, it is conceivable that the girls were rating their competence more favorably because they related more directly to the items measuring perceptions of physical competence. This interpretation is supported by the findings of Mantzicopolous [36], who found two item-level gender-related differences when administering the perception of physical competence sub-scale with young children. Across the developmental period of preschool to grade 2, Mantzicopolous found boys' perceptions of their climbing ability was consistently higher than the girls' and girls provided higher evaluations of their ability to skip. Further exploration of the items used to assess boys' and girls' perceptions of physical competence seems warranted. It may also be useful to consider the inclusion of items reflecting object control skills when investigating relationships with children's gross motor abilities.

We hypothesized that there would be no relationship between children's motor proficiency and their perceptions of physical competence. However, our data indicate a modest, but significant, relationship between motor proficiency and perceptions of physical competence. These relationships also appear to be gender specific. Regardless of motor skill type, boys with greater proficiency had higher perceptions of their physical competence. Girls' perceptions of physical competence were related to their locomotor skill proficiency, but not to their proficiency with object control skills. As stated earlier, it is possible that the girls related more to the actual test items of the perceptions of physical competence sub-scale and it also conceivable that girls discounted the importance of object control skills. Because we assessed children's perceptions of physical competence following motor skill testing, it is also possible that the three locomotor skills assessed in both measures (run, hop, and skip) were more tangible for the children. This may have affected the children's perceptions of competence. Future research could control this threat by assessing perceptions before skills.

The motor skills of this cohort of kindergarten children were low and proficiency varied by gender. Although motor skill levels were low, the children generally held positive perceptions of their physical competence. These positive perceptions provide a window of opportunity for learning and mastering motor skills. While young children hold a largely undifferentiated view of ability and effort they may be more predisposed to engage in, and persist with, activities that will foster their skillfulness. Although perceptions were generally high, we did find a modest relationship between perceptions of competence and motor skill proficiency. This finding suggests that children in kindergarten are already beginning to make self-judgments; and that affording opportunities to help them become and feel physically competent needs to occur early in their school, or preschool, life.

## Competing interests

The authors declare that they have no competing interests.

## Authors' contributions

ML drafted the manuscript; and participated in the data acquisition, data analysis and interpretation. LG and ES participated in data acquisition and interpretation and reviewed the manuscript. BW, RB, and PJN contributed to the design and conduct of the study, participated in data acquisition, and reviewed/revised the manuscript. VT contributed to the conception and design of the study; participated in data acquisition; was a significant manuscript writer; and conducted the data analysis. All authors read and approved the final manuscript.

## References

[B1] Janssen I Physical activity guidelines for children and youth Can J Public Health 2007 98 Suppl 2 S109 S121 18213942

[B2] Barnett LM van Beurden E Morgan PJ Brooks LO Beard JR Childhood motor skill proficiency as a predictor of adolescent physical activity J Adolesc Health 2008 44 252 259 19237111 10.1016/j.jadohealth.2008.07.004

[B3] Barnett LM van Beurden E Morgan PJ Brooks LO Beard JR Does childhood motor skill proficiency predict adolescent fitness? Med Sci Sports Exerc 2008 40 2137 2144 10.1249/MSS.0b013e31818160d3 18981934

[B4] Okley AD Booth ML Patterson JW Relationship of physical activity to fundamental movement skills among adolescents Med Sci Sports Exerc 2001 33 1899 1904 10.1097/00005768-200111000-00015 11689741

[B5] Raudsepp L Päll P The relationship between fundamental motor skills and outside-school physical activity of elementary school children Pediatr Exerc Sci 2006 18 426 435 10.1123/pes.18.4.426 39152604

[B6] Fisher A Reilly JJ Kelly LA Montgomery C Williamson A Paton JY Grant S Fundamental movement skills and habitual physical activity in young children Med Sci Sports Exerc 2005 37 684 688 10.1249/01.MSS.0000159138.48107.7D 15809570

[B7] Williams HG Pfeiffer KA O'Neill JR Dowda M McIver KL Brown WH Pate RR Motor skill performance and physical activity in preschool children Obesity 2008 16 1421 1426 10.1038/oby.2008.214 18388895

[B8] Lubans DR Morgan PJ Cliff DP Barnett LM Okely AD Fundamental movement skills in children and adolescents: Review of associated health benefits Sports Med 2010 40 1019 1035 10.2165/11536850-000000000-00000 21058749

[B9] Wrotniak BH Epstein LH Dorn JM Jones KE Kondilis VA The relationship between motor proficiency and physical activity in children Pediatrics 2006 118 e1758 e1765 10.1542/peds.2006-0742 17142498

[B10] Cantell MH Smyth MM Ahonen TP Clumsiness in adolescence: Educational, motor, and social outcomes of motor delay detected at 5 years Adapt Phys Act Quart 1994 11 115 129

[B11] Rose B Larkin D Berger BG Coordination and gender influences on the perceived competence of children Adapt Phys Act Quart 1997 14 210 221

[B12] Watson L Knott F Self-esteem and coping in children with developmental coordination disorder Br J Occup Ther 2006 69 450 456

[B13] Cumming SP Standage M Loney T Gammon C Neville H Sherar LB Malina RM The mediating role of physical self-concept on relations between biological maturity status and physical activity in adolescent females J Adolesc 2011 34 465 473 10.1016/j.adolescence.2010.06.006 20655102

[B14] Barnett LM Morgan PJ van Beurden E Beard JR Perceived sport competence mediates the relationship between childhood motor skill proficiency and adolescent physical activity and fitness: A longitudinal assessment Int J Behav Nutr Phys Act 2008 5 1 12 10.1186/1479-5868-5-1 18687148 PMC2569960

[B15] Stodden DF Goodway JD Langendorfer SJ Roberton MA Rudisill ME Garcia C Garcia LE A developmental perspective on the role of motor skill competence in physical activity: An emergent relationship Quest 2008 60 290 306 10.1080/00336297.2008.10483582

[B16] Horn T Weiss MR, Morgantown W Developmental Perspectives on Self-Perceptions in Children and Adolescents Developmental sport and exercise psychology: a lifespan perspective 2004 Va: Fitness Information Technology 101 143

[B17] Harter S Pike R Pictorial scale of perceived competence and social acceptance for young children Child Dev 1984 55 1969 1982 10.2307/1129772 6525886

[B18] Goodway JD Rudisill ME Perceived physical competence and actual motor skill competence of African American preschool children Adapt Phys Act Quart 1997 14 314 326

[B19] Goodway JD Crowe H Ward P Effects of motor skill instruction of fundamental motor skill development Adapt Phys Act Quart 2003 20 298 314

[B20] Goodway JD Suminski R Ruiz A The influence of project SKILL on the motor skill development of young disadvantaged Hispanic children. (Abstract) Res Q Exerc Sport 2003 74 A12 13

[B21] Hamilton M Goodway J Haubenstricker J Parent-assisted instruction in a motor skill program for at-risk preschool children Adapt Phys Act Quart 1999 16 415 426

[B22] Goodway JD Rudisill ME Influence of a motor skill intervention program on perceived competence of at-risk African American preschoolers Adapt Phys Act Quart 1996 13 288 301

[B23] Andersen L Sardinha L Froberg K Riddoch C Page A Anderssen S Fitness, fatness and clustering of cardiovascular risk factors in children from Denmark, Estonia and Portugal: The European Youth Heart Study Pediatr Obes 2008 3 58 66 10.1080/17477160801896366 18278634

[B24] Cardon G Van Cauwenberghe E Labarque V Haerens L De Bourdeaudhuij I The contribution of preschool playground factors in explaining children"s physical activity during recess Int J Behav Nutr Phys Act 2008 5 1 6 10.1186/1479-5868-5-1 18302741 PMC2266778

[B25] Pfeiffer KA Dowda M McIver KL Pate RR Factors related to objectively measured physical activity in preschool children Pediatr Exerc Sci 2009 21 196 208 19556625 10.1123/pes.21.2.196 PMC2719833

[B26] Robinson LE The relationship between perceived physical competence and fundamental motor skills in preschool children Child Care Health Dev 2010 37 589 596 21143273 10.1111/j.1365-2214.2010.01187.x

[B27] Beurden EV Zask A Barnett LM Dietrich UC Fundamental movement skills. How do primary school children perform? The 'Move it Groove it' program in rural Australia J Sci Med Sport 2002 5 244 252 10.1016/S1440-2440(02)80010-X 12413042

[B28] Ulrich DA Test of Gross Motor Development (TGMD-2) 2000 2 Austin: PRO-ED, Inc

[B29] Jacobs JE Lanza S Osgood DW Eccles JS Wigfield A Changes in children's self-competence and values: Gender and domain differences across grades one through twelve Child Dev 2002 73 509 10.1111/1467-8624.00421 11949906

[B30] Kershaw P Irwin L Trafford K Hertzman C The British Columbia atlas of child development 2005 Georgetown: Human Early Learning Partnership

[B31] The Human Early Learning Partnership EDI Data tables 2011 Vancouver: University of British Columbia

[B32] Harter S Manual for the self-perception profile for children. Revision of the Perceived Competence Scale for Children 1985 Denver: Unpublished manuscript, University of Denver

[B33] Baumgartner TA Strong CH Hensley LD Conducting and reading research in health and human performance 2002 New York: McGraw Hill

[B34] Neal R King P Comparison of a multivariate and a configural analysis for classifying engineering students J Couns Psychol 1969 16 563 568

[B35] Hardy LL King L Farrell L Macniven R Howlett S Fundamental movement skills among Australian preschool children J Sci Med Sport 2010 13 503 508 10.1016/j.jsams.2009.05.010 19850520

[B36] Mantzicopoulos P Younger children's changing self-concepts: boys and girls from preschool through second grade J Genet Psychol 2006 167 289 308 10.3200/GNTP.167.3.289-308 17278417

